# Tobacco Use and Cessation Counseling among Dental Professionals in Saudi Arabia

**DOI:** 10.3390/ijerph192316306

**Published:** 2022-12-06

**Authors:** Hussain Aljubran, Mohammed Alkoudemi, Mustafa Althunayan, Abdulrahman Aljeraisy, Amal Asiri, Muhammad Nazir

**Affiliations:** 1 College of Dentistry, Imam Abdulrahman Bin Faisal University, Dammam 31441, Saudi Arabia; 2 Department of Dental Education, College of Dentistry, Imam Abdulrahman Bin Faisal University, Dammam 31441, Saudi Arabia; 3 Department of Preventive Dental Sciences, College of Dentistry, Imam Abdulrahman Bin Faisal University, Dammam 31441, Saudi Arabia

**Keywords:** smoking, dentists, quitting, tobacco products

## Abstract

The study aimed to assess the prevalence of tobacco use among dental professionals and their attitudes and behaviors about tobacco cessation counseling in Saudi Arabia. A total of 359 male and female dental professionals participated in this cross-sectional study. A pretested self-administered questionnaire was administered among study participants by visiting dental hospitals and clinics in Saudi Arabia. In the study, 15.9% of the participants were current tobacco users and 50.4% had a tobacco user at home or the workplace. Most participants reported that tobacco use is associated with bad breath (88.9%), oral cancer (88.3%), dryness of the mouth (80.8%), and bad taste (79.9%). Most participants asked patients about their tobacco habits (86.6%), mentioned tobacco habits in patient records (71.3%), and explained the benefits of quitting tobacco (79.9%). Less than half the sample (47.1%) referred patients to tobacco quitting services/programs. Male gender (Adjusted odds ratio (AOR) 5.50, 95% CI 2.48–12.23, *p* < 0.001), having a tobacco user at home/the workplace (AOR 3.18, 95% CI 1.57–6.45, *p* 0.001), and believing that tobacco cessation counseling was a waste of time (AOR 2.32, 95% CI 1.13–4.76, *p* 0.021) were associated with significantly increased odds of tobacco use among dental professionals. The study concluded that tobacco use was common among dental professionals despite their awareness of the adverse effects of tobacco. Reduction in tobacco use and promotion of counseling skills among dental professionals should be achieved through public health measures.

## 1. Introduction

Tobacco use is a significant risk factor for several oral and systemic conditions [1]. According to the World Health Organization (WHO), there were 1.337 billion tobacco users globally in 2018 [2]. Causing more than 8 million deaths per year, tobacco is the most important health threat the world has ever encountered [3]. In Saudi Arabia, statistics by the Ministry of Health (2015) revealed that more than 3.45 million Saudis were smokers, and 24.9% of those were males and 1.7% were females [1]. These numbers are alarming due to the severe adverse effects of smoking on the human body. According to the Centers for Disease Control and Prevention, smoking increases the risk of coronary heart disease and stroke by 2–4 times and lung cancer in men by 25 times [4]. It is also a risk factor for type 2 diabetes, tuberculosis, and psychological disturbances [5].

There is a direct link between smoking and periodontal diseases such as gingivitis and periodontitis [6]. Smoking is also responsible for causing discoloration of teeth and restorations, dry sockets, tooth implant failure, and disturbs tissue healing [7]. Moreover, several cancers and precancerous lesions such as leukoplakia and leukokeratosis nicotine palatinus are associated with tobacco use which shows their early-stage manifestations in the oral cavity [6]. Therefore, dentists should be able to detect any clinical changes caused by smoking in the mouth [8].

Tobacco Cessation Counseling (TCC) provided by dentists can prevent the incidence of systemic and oral diseases and promote a healthier lifestyle for the patient [8]. By knowing the adverse effects of smoking and techniques of TCC, dental practitioners can educate and help their patients quit smoking and prevent its complications. There is also evidence of significantly lower odds of smoking associated with the awareness of oral complications of smoking [9]. Most dentists in the U.K. recognized their important role in TCC and they were willing to implement TCC strategies in their clinics [10]. Half of the dentists in Ontario, Canada expressed enthusiastic attitudes toward providing TCC to their patients [11]. In India, Bhat et al. found that the majority of dentists had positive attitudes towards TCC [12]. However, the situation can become worse when the dentists themselves turn out to be smokers. A study by Omana et al. showed that smoker dentists were less likely to implement smoking cessation techniques for their patients [8]. Dentists should have adequate knowledge and skills to practice TCC regardless of their smoking habits.

The oral cavity is frequently the first site to show negative health impacts from tobacco product use. Therefore, dentists as health care providers are in a leading position to identify adverse effects of tobacco on oral health and systemic health and help their patients quit smoking. A dentist’s role is important for providing patient support for tobacco cessation on a regular basis as they present for periodic dental examinations. This is especially beneficial for those individuals who are otherwise healthy and might not seek any medical care. This regularity also aids in maintaining abstinence from tobacco use or early detection of any relapse that might have occurred. Therefore, it is crucial to study the knowledge, attitudes, and practices related to tobacco use among dentists as health care providers. However, there is a lack of evidence about tobacco use among dentists in the Eastern Province of Saudi Arabia. Moreover, awareness of oral complications of tobacco and TCC techniques among dentists is not fully understood. Therefore, this study aimed to evaluate the prevalence of tobacco use among dental professionals, their awareness of the adverse effects of tobacco on oral health, and their attitudes and behavior toward TCC in the Eastern Province of Saudi Arabia.

## 2. Materials and Methods

This cross-sectional study was conducted on male and female dental professionals (general dentists, specialists, and consultants) working in the government and private sectors in Dammam, Khobar, Jubail, Qatif, Dhahran, Hofuf the Eastern Province of Saudi Arabia. The sample size calculations were based on (1) the population of dental professionals working in the Eastern Province (N = 2000), (2) the expected prevalence tobacco use among dental professionals (50%), (3) a 4% confidence limit, and a design effect equal to 1 [13]. These calculations produced a sample size of 462 dental professionals who were recruited using a convenience sampling technique.

The study questionnaire consisted of four parts. The first part included participants’ demographic information such as age, gender, place of work, qualifications, and monthly income. The second part included questions about dentists’ current tobacco use status and the presence of tobacco users at home or the workplace. Tobacco users included those who used cigarettes, smokeless tobacco, electronic cigarettes, and water pipes/Shisha. The third part was adopted from a previous study and included questions about dentist awareness of the adverse effects of tobacco use on oral health [9]. The fourth part was also derived from previous studies which investigated dentists’ attitudes towards TCC [14,15,16,17]. This part contained questions evaluating participants’ attitudes, perceived responsibility, and practices of TCC in their clinics. The questionnaire was pilot tested on 20 dentists to evaluate its feasibility, comprehensiveness, and time required for its completion. This process also helped further ensure the validity of the instrument. Data collected from this pilot test were not included in the main results of the study.

Ethical approval was also obtained from Imam Abdulrahman Bin Faisal University, Dammam. Participants were informed about the details of the study including their rights of voluntary participation. The administration of an anonymous questionnaire ensured the privacy and confidentiality of dentists’ responses. The dentists who provided written informed consent received a paper-based self-administered questionnaire. Ethical guidelines of the Declaration of Helsinki were followed during the study. The questionnaires were distributed among 462 dental professionals in governmental hospitals and private clinics. The researchers of the study visited these settings to collect dentists’ responses. A maximum of three visits were made to achieve an adequate response rate. However, 359 participants returned completed questionnaires.

Questionnaire data were entered in an Excel sheet and then transported to Statistical Package for Social Sciences software (IBM SPSS Statistics for Windows, Version 22. Armonk, NY, USA: IBM Corp) for statistical analyses. Descriptive and analytical statistical analyses were performed. Descriptive analysis included frequencies, percentages, means, and standard deviations. Analytical analyses included the chi-square test and multiple logistic regression analysis. These analyses evaluated the association of demographic and other factors with the prevalence of tobacco use among dental professionals. Statistical significance was determined using a *p*-value ˂ 0.05. 

## 3. Results

Data of 359 participants were included in the analysis and the response rate of the study was 77.7%. The study included 51.5% of males, 51.3% of Saudis, 66.3% of general dentists, and 68.2% of private practitioners. About 15.9% of dental professionals were current tobacco users with cigarette being the most common tobacco product (7%) followed by waterpipe (Hishah) (4.2%). Half of the participants (50.4%) reported that they were exposed to secondhand smoking (SHS) by a tobacco user at home or the workplace (Table 1). 

**Figure 1:**
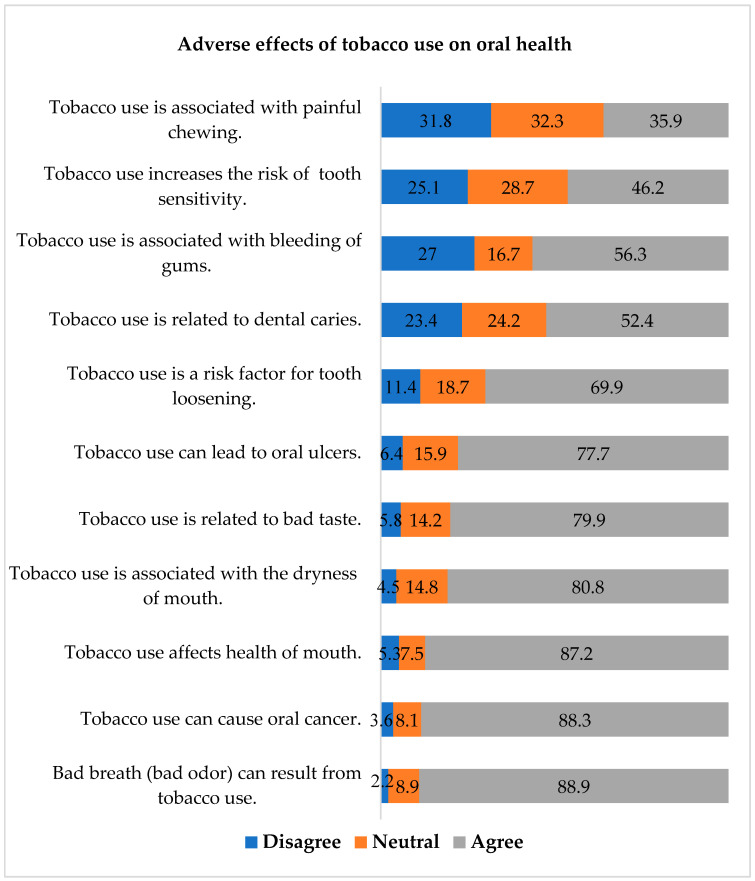
Dentists’ responses about the adverse effects of tobacco use on oral health.

Regarding the awareness of the adverse effects of tobacco on oral health, most participants reported that tobacco use was associated with bad breath (88.9%), oral cancer (88.3%), dryness of the mouth (80.8%), bad taste (79.9%), and oral ulcers (77.7%). Less than half of the sample reported that tobacco use was associated with painful chewing (35.9%) and an increased risk of tooth sensitivity (46.2%) (Figure 1).

**Figure 2:**
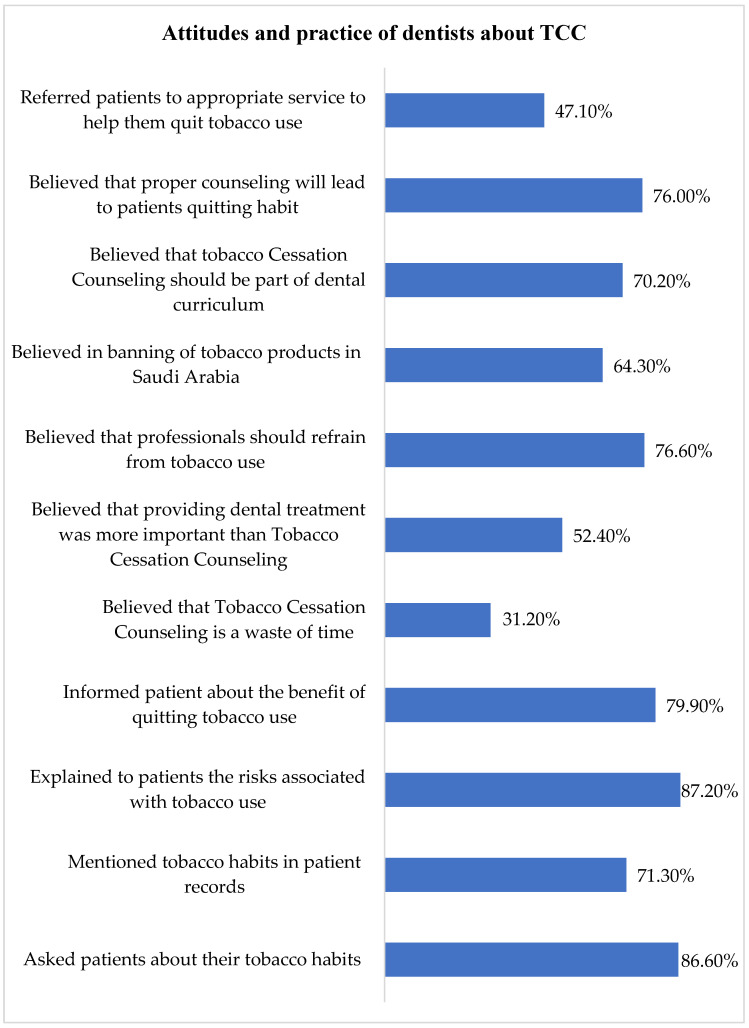
Distribution of dental professionals’ attitudes and behaviors about TCC.

The majority of participants reported asking their patients about tobacco habits (86.6%), mentioning tobacco use in patient records (71.3%), and explaining the benefits of quitting smoking (79.9%). TCC should be a part of the dental curriculum was suggested by 70.2% of the participants and 64.3% were in the favor of banning tobacco products in Saudi Arabia. Less than half the sample (47.1%) referred patients to services/programs to help them quit tobacco use. In the study, 47.1% of dental professionals reported that adequate TCC training was not available and only 17.7% were confident to a greater extent to effectively offer TCC technique to their patients (Figure 2). 

Bivariate analysis showed that male gender (OR 6.42, 95% CI 3.04–13.56, *p* < 0.001), having a tobacco user at home or the workplace (exposed to SHS) (OR 2.96, 95% CI 1.59–5.51, *p* < 0.001), believing that TCC was a waste of time (OR 1.93, 95% CI 1.08–3.45, *p* 0.024), and providing dental treatment was more important than TCC (OR 2.45, 95% CI 1.33–4.51, *p* 0.003) were associated with significantly increased odds of tobacco use among participants. On the other hand, belief in refraining from tobacco use (OR 0.26, 95% CI 0.14–0.47, *p* < 0.001), favoring tobacco ban (OR 0.26,95% CI 0.11–0.35, *p* < 0.001), and belief in TCC helping patient quick tobacco (OR 0.52, 95% CI 0.28–0.95, *p* 0.032) were significantly associated with a lower likelihood of tobacco use (Table 2). 

All variables used in bivariate analysis were entered in the final logistic regression model (Forward LR) and the results are shown in Table 3. The model showed that male gender (Adjusted odds ratio (AOR 5.50, 95% CI 2.48–12.23, *p* <0.001), having a tobacco user at home/workplace (exposed to SHS) (AOR 3.1895% CI 1.57–6.45, *p* 0.001), and believing that TCC was a waste of time (AOR 2.32, 95% CI 1.13–4.76, *p* 0.021) were significantly associated with increased odds of tobacco use among dental professionals. However, belief in banning tobacco products in the country was significantly associated with a lower likelihood of tobacco use (AOR 0.15, 95% CI 0.07–0.31, *p* < 0.001). 

## 4. Discussion

The present study evaluated the prevalence of tobacco use among dental professionals in the Eastern Province of Saudi Arabia and showed that 15.9% of participants were current tobacco users. The results of earlier local and regional studies showed a higher prevalence of tobacco use among dental professionals [15,18,19]. Alblowi reported that 23.8% of dental practitioners were current smokers in Jeddah, Saudi Arabia [18]. Alajmi et al. conducted a study on dentists in Kuwait and Saudi Arabia and showed that 27% of participants used one or more tobacco products [19]. A study by Bangera et al. showed that the prevalence of tobacco use was 43.3% among dentists in United Arab Emirates [15]. According to a study by Al-Maweri et al., 20% of dental professionals were current smokers in Yemen [16]. High awareness of the adverse effects of tobacco and the inclusion of 48.5% of females may account for the lower use of tobacco in the present study compared with other studies in Saudi Arabia. In a previous Japanese study, a high prevalence of smoking (27.1%) was shown among dentists [20]. On the other hand, data from other developed countries showed low smoking among dentists in Ireland (9%) [21], Norway (7%) [22], and Australia (3.9%) [23].

Male dental professionals were 5.5 times more likely to use tobacco products compared with their female counterparts in the present study. A previous similar study showed that more male dentists (37.1%) were smokers than female dentists (11.1%) in Saudi Arabia and Kuwait [19]. Similar results were shown in a study of dentists in Japan where 27.1% of participants were males compared with 3.4% of females [20]. The lower prevalence of tobacco use among female dentists in the present study is due to the cultural, religious, and social norms in the Middle East. In addition, female dentists are less likely to disclose tobacco use than male dentists [24].

Half of the sample in the present study reported the presence of a tobacco user at home or the workplace. The study also found that dental professionals who had a tobacco user at home or the workplace were about three times more likely to use tobacco than those without a tobacco user. The behavior of using tobacco is affected by a broad and complex range of influences, and social influences of family members and colleagues are established in the literature [25]. However, this is the first study to report the adjusted odds of tobacco use among dental professionals associated with family members or dental staff. These findings highlight the importance of holistic public health strategies aiming at the prevention of tobacco use and its burden among dental professionals, the dental team, and their family members. 

Dentists should play beyond their conventional role of patient oral care to address their personal and patients’ general health and quality of life needs [19]. They have an ethical and professional responsibility to encourage their patients to quit the consumption of tobacco products [26]. According to the WHO Global Oral Health Programme, dental professionals can play an important role in providing TCC to their patients and should include TCC in their routine dental checkups and treatment plans [27]. Therefore, they have an obligation to keep updated with current literature related to tobacco and emerging smoking habits or trends that may influence them and their patients [19].

The findings of the present study revealed that dental professionals have a high awareness of the adverse effects of tobacco use and the majority (79.9%) explained the benefits of quitting tobacco to their patients. In contrast, previous studies reported that 37% of dentists in the United Arab Emirates [15] and 57.1% of dentists in Nigeria [28] explained the benefits of quitting tobacco to their patients. Most dental professionals (64.3%) in the present study were also in favor of banning tobacco products in Saudi Arabia. According to WHO, the protocol aiming to end the illicit trade in tobacco products is the main option for reducing tobacco use and its negative effects on health and the economy. The tobacco epidemic is fueled by illicit trade, which makes tobacco products more widely available and more affordable while weakening tobacco control laws. In the past 5 years, the Saudi Arabian government has issued multiple laws and regulations to control and reduce tobacco sales and usage including, annual fees to be paid by any venue serving tobacco products and 100% taxation on the sale of tobacco products in recreational venues [2].

Interestingly, the dental professionals who were in favor of banning tobacco products were less likely to use tobacco than those who were against the tobacco ban in the country. This finding is understandable and underscores the importance of raising awareness about banning tobacco products to achieve a reduction in tobacco use among dental professionals. 

The lack of TCC training provided to dental professionals in Saudi Arabia is depicted in the present study where less than half of the sample reported the non-availability of adequate training in TCC. In addition, only 17.7% of dental professionals in our study were confident to a greater extent to effectively offer the TCC technique. The dental professionals in the central region (59.2%) [29] and the western region (50.8%) [30] of Saudi Arabia also reported low confidence in providing TCC, while only 10% of Kuwaiti dentists were very confident about their TCC skills [31]. It is known that a lack of training in TCC is a significant barrier to providing TCC to patients [32]. In contrast, Swedish dental practitioners were involved in several TCC initiatives after receiving formal training in tobacco control and prevention [33].

The literature indicates that most dentists consider TCC as an ineffective technique in dental practice [34]. Similar trends were observed in the present study where 31.20% of participants believed that TCC in dental clinics was a waste of time and 52.4% believed that providing dental treatment was more important than TCC. Even a greater proportion of dentists (55.8%) in India believed that TCC was a waste of time and that dental treatment was more important than TCC [17]. In Yemen, 50% of dental professionals believed that providing dental treatment was more important than TCC which may negatively influence their clinical practice and patient attendance [16]. On the other hand, only 18.1% of medical and dental interns in India believed that TCC was a waste of time [35]. This is an alarming finding which might negatively impact dentists’ TCC practices toward their patients. This might explain why 76% of the dentists involved in the study believed that proper TCC will lead patients to quit tobacco, yet only 47.10% referred patients to the relevant TCC services. Proper TCC education and training could potentially aid in alleviating this phenomenon and may also have the added benefit of lowering the incidence of tobacco use amongst dentists as health care providers and role models to their patients.

The present study has certain limitations. The study findings may not be generalized to all dental professionals working in Saudi Arabia because data represent responses of dental professionals from the Eastern Province. It was shown that self-reported smoking data were accurate in most studies included in a systematic review [24]. However, the under-reporting of responses in questionnaire-based studies should be considered while interpreting study findings. For instance, dental professionals may under-report tobacco use and may over-report TCC because of social desirability bias. Three visits were made to collect data from dental professionals to achieve a 90% or more response rate in the study. However, the study included data of 77.7% of dental professionals. Some questionnaires were discarded due to incomplete or missing information and some dentists did not fill up the survey due to their busy schedules. The present study reported the proportion of tobacco users at home or the workplace and their influence on tobacco use patterns among dental professionals. However, further information related to tobacco use among dental staff and family members was not sought in the present study. In the future, a large nationwide study should investigate the role of the dental team and their family members in tobacco use among dental professionals.

## 5. Conclusions

The study concluded that tobacco use was common among dental professionals despite their awareness of the adverse effects of tobacco. Male dental professionals were five times more likely to use tobacco products compared to female dental professionals. Having a tobacco user at home or the workplace resulted in a greater likelihood of dental professionals using tobacco products. The majority of dental professionals believed in banning tobacco products in Saudi Arabia but reported low confidence in providing TCC to their patients. Reduction in tobacco use and promotion of counseling skills among dental professionals should be achieved through holistic public health approaches.

## Figures and Tables

**Table 1:** Characteristics of study participants (N = 359).

Study Variables	N (%)
**Gender**	
Male	185 (51.5)
Female	174 (48.5)
**Nationality**	
Saudi	184 (51.3)
Non-Saudi	175 (48.7)
**Place of work**	
Government sector	114 (31.8)
Private sector	245 (68.2)
**Qualifications**	
General Dentist	238 (66.3)
Specialist/Consultant	121 (33.7)
**Income (Saudi Riyal)**	
<10,000 per month	142 (39.6)
≥10,000 per month	217 (60.4)
**Marital Status**	
Single	129 (35.9)
Married	230 (64.1)
**Years in dental practice**	
<10 years	196 (54.6)
≥10 years	163 (45.4)
**Current tobacco user**	
Cigarette	57 (15.9)
Cigars	25 (7)
Smokeless tobacco	7 (1.9)
Electronic Cigarettes	10 (2.8)
Waterpipe (Hishah)	15 (4.2)
**Had a tobacco user at home/workplace (exposed to SHS)**	181 (50.4)

**Table 2:** Bivariate Analysis: Tobacco use among dental professionals and its relationship with study variables.

Variables	Unadjusted Odds Ratio (95% CI)	*p*-Value
**Gender**	6.42 (3.04,13.56)	<0.001 *
Male		
Female (Reference category)		
**Nationality**	1.16 (0.66, 2.05)	0.606
Saudi		
Non-Saudi (Reference category)		
**Place of work**	0.73 (0.39, 1.38)	0.336
Government sector		
Private sector (Reference category)		
**Qualifications**	1.24 (0.67, 2.29)	0.499
General Dentist		
Specialist/Consultant (Reference category)		
**Income (Saudi Riyal)**	0.73 (0.40, 1.32)	0.295
<10,000 per month		
≥10,000 per month (Reference category)		
**Marital Status**	1.36 (0.77, 2.43)	0.290
Single		
Married (Reference category)		
**Years in dental practice**	0.84 (0.47, 1.48)	0.539
<10 years		
≥10 years (Reference category)		
**Had a tobacco user at home/workplace (exposed to SHS)**	2.96 (1.59, 5.51)	<0.001 *
Yes		
No (Reference category)		
**Believed that TCC is a waste of time.**	1.93 (1.08, 3.45)	0.024 *
Yes		
No (Reference category)		
**Believed that providing dental treatment was more important than TCC.**	2.45 (1.33, 4.51)	0.003 *
Yes		
No (Reference category)		
**Believed that dental professionals should refrain from tobacco use**	0.26 (0.14, 0.47)	<0.001 *
Yes		
No (Reference category)		
**Favored banning of tobacco products in Saudi Arabia.**	0.19 (0.11, 0.35)	<0.001 *
Yes		
No (Reference category)		
**Believed that proper TCC will lead patients to quit tobacco.**	0.52 (0.28, 0.95)	0.032 *
Yes		
No (Reference category)		

* statistically significant.

**Table 3:** Multiple logistic regression final model. Tobacco use among dental professionals and its relationship with study variables.

Variables	β	S.E	Adjusted Odds Ratio (95% CI)	*p*-Value
**Gender**	1.705	0.407	5.50 (2.48, 12.23)	<0.001
Male				
Female (Reference category)				
**Had a tobacco user at home/workplace (exposed to SHS)**	1.156	0.361	3.18 (1.57, 6.45)	0.001
Yes				
No (Reference category)				
**Believed that TCC is a waste of time**	0.843	0.366	2.32 (1.13, 4.76)	0.021
Yes				
No (Reference category)				
**Believed that providing dental treatment was more important than TCC**	0.927	0.369	2.53 (1.23, 5.21)	0.012
Yes				
No (Reference category)				
**Believed in banning of tobacco products in Saudi Arabia**	−1.881	0.363	0.15 (0.07, 0.31)	<0.001
Yes				
No (Reference category)				

## Data Availability

The data can be obtained from the corresponding author on reasonable request.
